# Human Papillomavirus Vaccination in Pediatric, Adolescent, and Young Adult Cancer Survivors—Opportunity to Address Gaps in Cancer Prevention and Survivorship

**DOI:** 10.3390/vaccines12020114

**Published:** 2024-01-24

**Authors:** Melissa A. Kluczynski, Elisa M. Rodriguez, Cailey S. McGillicuddy, Nicolas F. Schlecht

**Affiliations:** Department of Cancer Prevention and Control, Roswell Park Comprehensive Cancer Center, Elm & Carlton Streets, Buffalo, NY 14263, USA; melissa.kluczynski@roswellpark.org (M.A.K.); elisa.rodriguez@roswellpark.org (E.M.R.); cailey.mcgillicuddy@roswellpark.org (C.S.M.)

**Keywords:** human papillomavirus (HPV) vaccine, survivors of childhood cancer, pediatric, adolescent, and young adult, cancer survivors, secondary cancers, transition of care, survivorship plan, secondary cancer prevention

## Abstract

The risks of secondary cancers associated with human papillomavirus (HPV) infection are as much as three times higher for survivors of pediatric, adolescent, and young adult cancer (PYAC) compared to the general population. Despite this, HPV vaccination rates among PYAC survivors remain low. Whereas pediatric oncology providers endorse HPV vaccination of PYAC survivors, many lack the resources or opportunities to intervene. The responsibility of HPV vaccination, therefore, falls to primary care providers and practices. This article provides an overview of the challenges with HPV vaccination that are distinct to PYAC survivors and discusses potential strategies to increase HPV vaccine coverage in this population.

## 1. Introduction

The number of pediatric, adolescent, and young adult cancer (PYAC) survivors has grown considerably thanks to recent advancements in treatment. However, PYAC survivors are at increased risk for infections and secondary malignancies due to the long-lasting immunosuppressive effects of cancer therapy (i.e., chemotherapy, radiation, immunotherapy, and bone marrow transplantation) [1,2,3,4,5,6]. Human papillomavirus (HPV) infections are the most common sexually transmitted infection in the U.S. and the central etiologic factor for almost all cervical cancers and most anal, oropharyngeal, and genital cancers [7,8,9,10].

Recent national estimates of HPV prevalence indicate a substantial proportion of the U.S. population (22.0%), including 24.2% of men and 19.9% of women 15–59 years of age, continue to harbor disease-associated HPV infections [11]. More than 46,000 new cases of HPV-related cancers occur annually in the U.S., of which the most common are cervical cancer in women and oropharyngeal cancer in men [12]. Over 4000 deaths from cervical cancer and 10,000 deaths from oropharyngeal cancers occur each year in the U.S. [13]. Globally, the incidence of new cancer cases that were attributable to HPV was 8 cases per 100,000 person-years, or 690,000 new cases, of which most were cervical cancer [14]. Compared to the general population, the rates of HPV-related cancers (particularly anal, oropharyngeal, and genital cancers) are higher among PYAC survivors; Ojha et al. found rates were 40% higher for female and 150% higher for male PYAC survivors, while more recently, Henderson et al. reported a nearly threefold increased incidence among all cancer survivors [6,15].

Prophylactic HPV vaccines were initially approved more than a decade ago and have shown high efficacy against HPV infection and associated neoplasia, demonstrated by a 64% drop in prevalence in vaccine types among females aged 14–19 years and a 34% decrease among those aged 20–24 years in the U.S. In addition, recent evidence also suggests that rates of cervical precancer and hospitalizations for HPV-related disease have declined in the U.S. since the introduction of the HPV vaccines [16,17]. It is estimated that 70% of oropharyngeal cancers are preventable with HPV vaccination, in addition to 90% of cervical and anal cancers, 60–70% of vaginal and vulvar cancers, and 70% of penile cancers [18,19,20]. Vaccination is imperative for preventing HPV-related disease because, aside from cervical and anal cancer screening tests (e.g., Papanicolaou (Pap) test, HPV DNA tests), there is a lack of screening tools for other types of HPV-related cancers [7]. However, HPV vaccination rates in the U.S. remain suboptimal, attributed to missed opportunities for vaccination (i.e., health visits during which at least one vaccine, other than the HPV vaccine, is received) [21,22,23]. The Centers for Disease Control and Prevention (CDC) estimated that if the HPV vaccine was administered to all adolescent girls born in the year 2000 during health visits when they received another vaccine, HPV vaccine initiation by age 13 years could have reached 91.3% [24].

HPV vaccination is currently recommended by the CDC for males and females aged 9 to 26 years as a two-dose series for persons vaccinated before age 15 years and a three-dose series for those vaccinated at age 15 years or older, immunocompromised persons and individuals with malignant neoplasms [25]. The HPV vaccine can also be co-administered safely with other age-appropriate vaccines (e.g., tetanus, diphtheria and pertussis (Tdap) and meningococcal) [26]. While the HPV vaccine has been demonstrated to be safe for use among PYAC survivors [27], vaccine efficacy may be reduced among individuals already infected with HPV and among immunocompromised individuals [28,29].

The current HPV vaccines available to the general population have been shown to be safe and effective for cancer survivors [27], and the CDC and Children’s Oncology Group (COG) recommend vaccinating patients within 6 months after cancer treatment [25]. Despite this, HPV vaccination rates among young cancer survivors remain low; compared to the general population, HPV vaccination rates are as much as 20% lower for PYAC survivors and are well below the 80% Healthy People 2030 target [23,30,31,32,33]. A recent review of five cancer centers reported that only 24% of cancer survivors have initiated HPV vaccination, with just 13.5% completing the vaccine series [31]. In addition, a recent study at our cancer center showed that less than half of pediatric cancer survivors received at least one dose after five years, and less than a quarter completed all required doses in the vaccine series [23].

Antibody levels for vaccine-preventable diseases tend to diminish in PYAC survivors after cancer treatment, likely due to the genotoxic effects of cancer therapy itself (e.g., pelvic irradiation) and/or prolonged immunosuppression, subsequently increasing the risk for oncogenic infections such as HPV [34,35,36]. This further emphasizes the need for improving HPV vaccination rates among PYAC survivors, including the potential need for booster doses if initial doses were received before cancer therapy [37,38,39,40]. Whereas pediatric oncology providers endorse the HPV vaccination of young cancer survivors, many lack the resources or opportunities to intervene [41]. The responsibility of the HPV vaccination of PYAC survivors, therefore, falls to primary care providers (PCPs), but remains suboptimal, as reflected by the low vaccination rates [23,31,32,33]. This article provides a brief overview of the challenges and opportunities associated with increasing HPV vaccine coverage among PYAC survivors.

## 2. Barriers to HPV Vaccination

Provider recommendation was found to be among the strongest predictors of vaccine initiation for young cancer survivors [31,42,43]. Unfortunately, only 28% of PYAC survivors report obtaining an HPV vaccine recommendation from their healthcare providers [31]. One study found a non-significant cancer-control difference in which a minority of survivor families reported receiving a provider recommendation for the HPV vaccine, whereas a majority of control families received a recommendation [44]. The authors suggested that future interventions target provider communication/recommendation because there may be confusion among providers as to whether the PCP or oncologist currently is or should be managing HPV vaccinations. The study also examined other factors for association with vaccine initiation and vaccine completion, including vaccine knowledge, vaccine communication, and health belief factors; they found that an older daughter age was positively associated with vaccine initiation, while the perception of high vaccine barriers and severity associated with HPV infection and complications was negatively associated with vaccine completion.

Many of the patient-/caregiver-reported and clinician-reported barriers to HPV vaccination for the general population [45,46,47,48,49,50,51,52,53,54,55,56,57,58,59,60,61,62,63,64,65,66,67] overlap with those reported for PYAC survivors [41,42,68,69,70,71], whereas some barriers are unique to the PYAC survivor population (Table 1). For instance, PYAC survivors and their caregivers reported concerns about the safety, side effects, and applicability of the HPV vaccine in relation to their cancer history and survivorship [41,68,72]. Cherven et al. discovered that cancer survivors exhibited a higher tendency to decline participation in an open-label trial investigating the immunogenicity and safety of the HPV vaccine due to concerns regarding its safety [72]. In a separate study conducted by Waters et al. on PYAC survivors and their caregivers, it was revealed that the majority of participants considered a recommendation for the HPV vaccine from a trusted healthcare provider as the decisive factor in their decision to pursue vaccination against HPV [68]. Moreover, the participants in the study expressed a preference for receiving the HPV vaccine recommendation from their oncologist, as they felt at ease with them and trusted their expertise in cancer care. Kirchhoff et al. also found that caregivers of PYAC survivors who received vaccine recommendations from their cancer care team were 35% more likely to have their child vaccinated against HPV compared to caregivers who did not receive such a recommendation [69].

Other barriers to receiving the vaccine include lack of education about HPV and HPV vaccination, parental concerns about behavioral consequences of vaccination such as fears of promiscuous sexual behavior, parental beliefs that their child is not at risk for HPV either because their child was too young or not sexually active, and the cost of the vaccine [45,48,52,56,68,69,73]. Whereas HPV vaccination has not been shown to increase sexual activity or accelerate sexual debut in adolescent and young adult men and women [74,75,76], studies found that PYAC survivors may engage in high-risk sexual behaviors, including earlier sexual debut, more sexual partners, and less frequent condom use due to perceived infertility, or increased cognitive behavioral issues (e.g., inattention and/or hyperactivity), ultimately increasing their risk for HPV acquisition [73,77]. One study found that despite perceiving high-risk sexual behavior as associated with increased susceptibility to HPV infection by young adult cancer survivors, most survivors (75%) did not receive the HPV vaccine [73].

Clinicians caring for PYAC survivors reported the following barriers to HPV vaccination: (a) uncertainty regarding which type of provider is responsible for administering vaccines (e.g., PCP versus oncologist); (b) lack of communication between PCPs and oncologists regarding cancer diagnosis, treatment, and follow-up care; and (c) unclear guidelines for administering the HPV vaccine to cancer survivors [41,70,71]. In one study, only 30% of PCPs felt confident in their knowledge about immunizations for childhood cancer survivors, which is concerning because PCPs may be missing opportunities for immunizing this vulnerable population [78]. Clinicians also reported concerns about vaccine safety, costs associated with providing the vaccine or low reimbursement rates, and beliefs that the HPV vaccine is less important than other vaccines [41,70,79,80]. Oncologists also report feeling overburdened by busy clinic schedules and difficulties in adjusting the workflow in oncology practice to be able to administer the HPV vaccine [41,70].

Although vaccination is not usually a priority in oncology practices, the oncology care team can play a critical role in recommending HPV vaccination to PYAC survivors since they can effectively discuss vaccine safety concerns following cancer therapy and refer survivors to a PCP who can administer the HPV vaccine [68]. As with the general population, providers treating PYAC survivors reported beliefs that patients or parents are vaccine-hesitant and about being uncomfortable discussing sexual topics [41,70,71,79]. However, reports that PYAC survivors are more likely to engage in high-risk sexual behaviors, as described above, highlight the importance of having a conversation about HPV vaccination with PYAC patients early [73,77]. How providers perceive parental vaccine hesitancy can be an important driver in their willingness to recommend the HPV vaccine, delivery of the recommendation, and responses when parents refuse the vaccine [81]. Most providers in our survey study [41] identified cancer prevention education on HPV vaccines as both an opportunity and responsibility of providers treating PYAC survivors, although provider-level HPV vaccine knowledge was also identified as a barrier. Unfortunately, there are few implementation science studies targeting cancer prevention strategies targeting PYAC survivors [82].

**Table 1:** HPV vaccination barriers reported in the literature by the general population and pediatric, adolescent, and young adult cancer survivors.

Barriers to HPV Vaccination	Studies Focused on the General Population	Studies Focused on PYAC Survivors
Patient-/Caregiver-Reported:		
- Lack of provider recommendation	[45,46,47,48,83]	[42,68,72,84,85]
- Lack of education/understanding about HPV and HPV vaccination	[45,49,50,51,52,53,54,83]	[68,69,72,79,84,85]
- Concerns about vaccine safety and adverse effects (including those related to survivorship)	[45,48,50,52,55,83]	[69,72]
- Parental concerns about behavioral consequences of vaccination (e.g., fears of promiscuity)	[52,56,83]	
- Parental beliefs their child is not at risk (e.g., too young, not sexually active, infertile)	[45,46,48,49,50,51,52,53,54,83]	[69,72,84]
- Parental concerns about cost	[45,48,52,83]	
Clinician-Reported:		
- Insufficient knowledge of HPV and HPV vaccination	[57,58,59,67,80]	[41,70,71,79,80]
- Concerns about vaccine safety, efficacy, and duration of immunity	[57,58,60,61,62,63]	[41,70,79,80]
- Perception that HPV vaccination is less important than other vaccines	[62,64,67]	[41,79,80]
- Perceived patient or parent vaccine hesitancy	[57,58,59,60,65]	[41,70,79]
- Discomfort communicating about sexual topics	[57,62,66]	[41,71,79]
- Clinic-related (e.g., lack of staff to administer vaccines, lack of storage for the vaccine)	[58,67,86]	[41,70,79,80]
- Financial issues (e.g., costs to provide vaccination/low reimbursement and lack of insurance coverage)	[57,58,59,60,63,65,67,86,87,88,89,90,91,92]	[41,79]
- Lack of efficient communication between primary care and oncology providers		[41,70,71,80]
- Uncertainty regarding who is responsible for vaccination (primary care vs. oncology provider)		[41,70]
- Unclear guidelines for administering vaccines to cancer survivors	[80]	[41,70,71,79,80]

HPV, human papillomavirus; PYAC, pediatric, adolescent, and young adult cancer.

## 3. Transition of Care—An Opportunity for Increasing HPV Vaccination Uptake

PYAC survivors require lifelong follow-up care for the management of late effects from cancer treatment and to obtain appropriate risk-based surveillance, such as screening for secondary cancer [93]. Most PYAC survivors will continue to receive follow-up care from a cancer center through the end of adolescence and transition to a PCP by early adulthood, although follow-up care attendance decreases over time [94,95,96]. A study by Oeffinger et al. found the older the PYAC survivor, the less likely they were to report having had a physical examination, cancer-related visit, or visit to a cancer center within the past two years [96]. For example, the percentage of PYAC survivors that reported attending a cancer-related visit within the past two years was 48.6% for 18- to 19-year-olds, 45.1% for 20- to 24-year-olds, 38.7% for 25- to 29-year-olds, and so forth. Cancer survivors engaged in regular follow-up care have greater improvement in health and educational outcomes, such as increased knowledge about their diagnosis, treatment, and risk for late effects; increased health surveillance; and increased detection of late effects compared to survivors that do not engage in regular follow-up care [97]. The transition of care may, therefore, provide an opportunity for increasing HPV vaccination uptake among PYAC survivors. However, methods for implementing HPV vaccination into survivorship care are lacking due to the absence of standard models of transitional care and barriers related to health care transition [41,94,98].

PYAC survivor-reported barriers to transition of care include dependence on pediatric providers, less confidence in PCPs, inadequate communication between patient and provider, and cognitive difficulty [41,99,100]. To optimize the transition experience, PYAC survivors report a preference for increased knowledge of late effects and the need for long-term follow-up care, increased support throughout the transition process, and improved communication and comfort levels between providers and survivors and their families [41,100,101,102]. PYAC survivors also prefer having their pediatric oncologist as their primary source of information because they have established a close and trusting relationship with them [100]. Therefore, pediatric oncologists are key players when it comes to educating PYAC survivors about HPV and recommending vaccination.

For pediatric oncologists, barriers to the transition of care include patient–provider emotional attachment, a lack of providers with young adult survivorship expertise, and the complex emotional, social, and/or medical needs of PYAC survivors [103]. For PCPs, barriers include not receiving patient-specific survivorship care plans (SCPs) from an oncologist, a lack of familiarity with survivorship guidelines, a lack of formal training in survivorship care, and a lack of time to adequately address survivorship issues [104,105,106,107,108]. Although many PCPs feel uncomfortable and unprepared for treating cancer survivors, most are willing to care for survivors if they can obtain a SCP and/or consult with a cancer center-based oncologist or survivorship program [105,106]. Thus, the multidisciplinary care of PYAC survivors is essential and requires increased knowledge, communication, and collaboration between pediatric oncologists and PCPs [70]. Teamwork among pediatric oncologists and PCPs is crucial when it comes to increasing HPV vaccination rates.

## 4. Implementing Practice-Level Interventions

Practice-level interventions that engage primary care teams in utilizing evidence-based strategies to increase adolescent HPV vaccination rates among the general population are demonstrated to be effective but have not been tested for PYAC survivors [67,109,110]. The following evidence-based strategies have been implemented successfully in practices to increase HPV vaccination: provider recommendations [111], provider and patient education [112,113,114], provider prompts [67,115,116], standing orders [67,116], patient reminder systems [67,112,116], and/or provider assessment and feedback [67,112,113,114,116]. Additionally, two intervention approaches (i.e., the 4 Pillars™ Practice Transformation Program and the Assessment, Feedback, Incentives, and eXchange (AFIX)) are effective at increasing adolescent vaccine coverage, including HPV, Tdap, and meningococcal vaccines [117,118]. Multicomponent interventions have the most impact on HPV vaccination coverage based on a systematic review by the Community Preventive Services Task Force, which concluded that provider assessment and feedback had positive effects on HPV vaccine initiation, whereas patient reminders had a positive effect on HPV vaccine completion [109].

The Community Guide provides information on evidence-based interventions to improve the coverage of vaccines recommended for routine use among children, adolescents, and adults and recommends the following provider-focused approaches [119]. Team huddles and performance evaluation can be effective for providing clinicians with the knowledge they need regarding HPV and HPV vaccination, resolving clinic issues like lack of staff or vaccine storage issues, improving communication between primary care and oncology providers, and reviewing guidelines for vaccine administration. The execution of standing orders for adolescent vaccines, including HPV vaccination, can reduce the need for provider recommendations and improve workflow in the clinic when it comes to administering vaccines. Introducing HPV vaccination early can reinforce recommendations from a provider, including by both the oncologist and PCP, and increase knowledge about HPV and the HPV vaccine. Incorporating these recommendations during the transition of care may help to address parental concerns regarding behavioral consequences of vaccination and believing their child is not at risk and make for an easier conversation between patients, caregivers, and clinicians regarding a sensitive topic. Furthermore, keeping the conversation focused on secondary cancer prevention education can also provide the needed education regarding HPV and HPV vaccination and minimize some parental concerns and hesitancy surrounding the vaccine. Provider HPV vaccination recommendations given in the same way and on the same day as other adolescent vaccines can aid in minimizing vaccine safety concerns, parental hesitancy, and concerns about vaccination cost [120,121]. Examples of how these interventions can address many of the barriers to HPV vaccination in PYAC survivors expressed by patients and PCPs are summarized in Table 2.

**Table 2:** Strategies to address HPV vaccination barriers among pediatric, adolescent, and young adult cancer survivors as reported by patients, caregivers, and clinicians.

Barriers to HPV Vaccination	Implementation Science-Focused Interventions				
Patient-/Caregiver-Reported:	Team Huddles and Performance Evaluation	Execution of Standing Orders	Introduce HPV Vaccination Early	Secondary Cancer Prevention Education	Recommendation for Vaccination Conducted in Same Way as Other Adolescent Vaccines
- Lack of provider recommendation		√	√		
- Lack of education/understanding about HPV and HPV vaccination			√	√	
- Concerns about vaccine safety and adverse effects			√		√
- Parental concerns about behavioral consequences of vaccination (e.g., fears of promiscuity)			√	√	
- Parental beliefs their child is not at risk (e.g., too young, not sexually active, infertile)			√	√	√
- Parental concerns about cost					√
Clinician-Reported:					
- Insufficient knowledge of HPV and HPV vaccination	√			√	
- Concerns about vaccine safety, efficacy, and duration of immunity	√		√	√	√
- Perception that HPV vaccination is less important than other vaccines				√	√
- Perceived patient or parent vaccine hesitancy			√	√	√
- Discomfort communicating about sexual topics			√	√	√
- Clinic-related (e.g., lack of staff to administer vaccines, lack of storage for the vaccine)	√	√			
- Financial issues (e.g., costs to provide vaccination/low reimbursement and lack of insurance coverage)					√
- Lack of efficient communication between primary care and oncology providers	√	√			
- Uncertainty regarding which provider is responsible for vaccination (primary care vs. oncology provider)	√	√			
- Unclear guidelines for administering vaccines to cancer survivors	√	√			√

HPV, human papillomavirus. Check marks (√) indicate which barriers are addressed by each implementation science-focused intervention.

## 5. Future Directions

Despite the effectiveness of practice-level interventions for increasing HPV vaccination rates [109,110], there is a dearth of interventions connecting cancer center services to primary care during the transition period, which represents a missed opportunity to reinforce clinical partnerships and increase HPV vaccination rates among PYAC survivors. Most interventions focus on practices within integrated health networks or specialized pediatric oncology clinics. One recent example includes a study by Landier et al. [98] that proposes to test a practice-level provider-focused intervention (HPV PROTECT) to increase HPV vaccine uptake among young cancer survivors seen in pediatric oncology clinics. The intervention incorporates some of the same components described in approaches to promote vaccination used in other populations, including provider communication training, assessment and peer feedback, and provider toolkits [112,113,114,116]. Two resources within HPV PROTECT’s provider toolkit relate to primary care—a vaccine action plan including primary care offices as potential vaccination locations and a standardized template for oncologist-PCP communication specific to the vaccination plan. However, this puts a primary focus on training oncology providers rather than on fostering collaboration between pediatric oncology centers and primary care practices where the majority of PYAC survivors return to following cancer treatment [96,122]. For these patients, transition of care models and effective communication between pediatric oncologists and PCPs become of primary import, highlighting the need for primary care practice focused interventions.

Lastly, research shows that parents and providers prefer a cancer prevention focus when recommending HPV vaccination [123]; however, the impact of HPV vaccine education and care coordination during the transition of care for PYAC patients remains unclear. Encouraging oncologists to recommend HPV vaccination, including emphasizing this in the COG follow-up guidelines [124], could help reinforce and improve vaccination rates among PYAC survivors. Leveraging patient education and implementing care coordination services into the transition of care at the cancer center/oncology practice level may be an efficient and effective approach for increasing HPV vaccination among PYAC survivors. This approach should be based on the established implementation of science-focused interventions in collaboration with primary care practices that consider community, culture, and health systems to increase adolescent HPV vaccination rates in PYAC survivors [125,126]. Furthermore, establishing transition-of-care models for increasing HPV vaccination may also serve as the framework for implementing strategies for promoting vaccines for other cancers caused by viruses as they become available [127].

## 6. Conclusions

PYAC survivors are at increased risk for HPV-related cancers. Despite this, HPV vaccination rates remain suboptimal for this population. While there is extensive research on the barriers and opportunities for HPV vaccination in the general population, there is little research on these topics in PYAC survivors. This article provides an overview of the challenges with HPV vaccination that are distinct to PYAC survivors and discusses potential strategies to increase HPV vaccine coverage in this population, including novel practice-level interventions for increasing vaccine uptake that can be integrated into transition of care models for PYAC survivors.

Most PYAC survivors do not receive a provider recommendation for HPV vaccination, thereby highlighting a gap in cancer prevention for this at-risk patient population. Furthermore, while pediatric oncology providers endorse HPV vaccination of young cancer survivors, many lack the resources or opportunities to intervene. The responsibility of HPV vaccination among PYAC survivors, therefore, falls to PCPs, but remains suboptimal, as reflected by the low vaccination rates. Transition of care models provided at the cancer center level that integrate evidence-based strategies for HPV immunization may offer an opportunity for increasing HPV vaccine uptake among PYAC survivors. However, interventions connecting the cancer center to primary care during the transition period following completion of cancer treatment among PYAC survivors are lacking.

## Abbreviations

Human papillomavirus (HPV); pediatric, adolescent, and young adult cancer (PYAC); Centers for Disease Control and Prevention (CDC); Children’s Oncology Group (COG); tetanus, diphtheria, and pertussis (Tdap); primary care provider (PCP); survivorship care plan (SCP); Assessment, Feedback, Incentives, and eXchange (AFIX); National Cancer Institute (NCI).
